# Retraction: 24-Hour Rhythms of DNA Methylation and Their Relation with Rhythms of RNA Expression in the Human Dorsolateral Prefrontal Cortex

**DOI:** 10.1371/journal.pgen.1009895

**Published:** 2021-11-04

**Authors:** Andrew S. P. Lim, Gyan P. Srivastava, Lei Yu, Lori B. Chibnik, Jishu Xu, Aron S. Buchman, Julie A. Schneider, Amanda J. Myers, David A. Bennett, Philip L. De Jager, the PLOS Genetics Editors

After publication of this article [1], concerns were raised regarding the validity of the conclusions. Specifically, the p-values used to support Fig 2 [1] are based on a permutation approach that was incorrectly reported, as outlined in [2], and, when repeated using a more robust approach, does not support the central conclusion regarding rhythmicity of methylation. In addition, comparison of nadir time distributions between groups as carried out for Fig 6 [1] is recognized to be insufficient to support conclusions about relationships between sex, age, Alzheimer’s disease, or 24-hour activity rhythms and the timing of rhythms of methylation.

The authors recognize the weaknesses present in the analytical approach used in this article that were uncovered after the paper was published; an inadvertent error led to results which were significant and specifically affects the results shown in Figs 2 and 6, and from which some of the conclusions of the article were derived.

In light of the above issues with the analytic approach and the concern that a number of key conclusions are not fully supported by the published analyses in [1], the authors and the *PLOS Genetics* Editors retract this article.

All authors agreed with the retraction.
